# COVID-19 infection: mitohormetic concept of immune response

**DOI:** 10.1038/s41420-020-00297-9

**Published:** 2020-07-14

**Authors:** Jerzy Gebicki, Marzena Wieczorkowska

**Affiliations:** 1 Institute of Applied Radiation Chemistry, Lodz University of Technology, 90-924 Lodz, Poland; 2 Pharmena, 90-530 Lodz, Poland

**Keywords:** Drug development, Respiratory distress syndrome

In the Editorial of a recent issue of *Cell Death & Differentiation*, the authors proposed that the immune response in COVID-19 has two phases: non-severe (immune protection) and severe (inflammation damaging)^1^. They suggest using Vitamin B3 (nicotinamide and nicotinic acid) in the second phase. Unless we have a clearer understanding of the mechanism of Vitamin B3 anti-inflammatory activity, particularly in lung tissue, it is difficult to progress toward an evaluation of this solution.

**Fig. 1:**
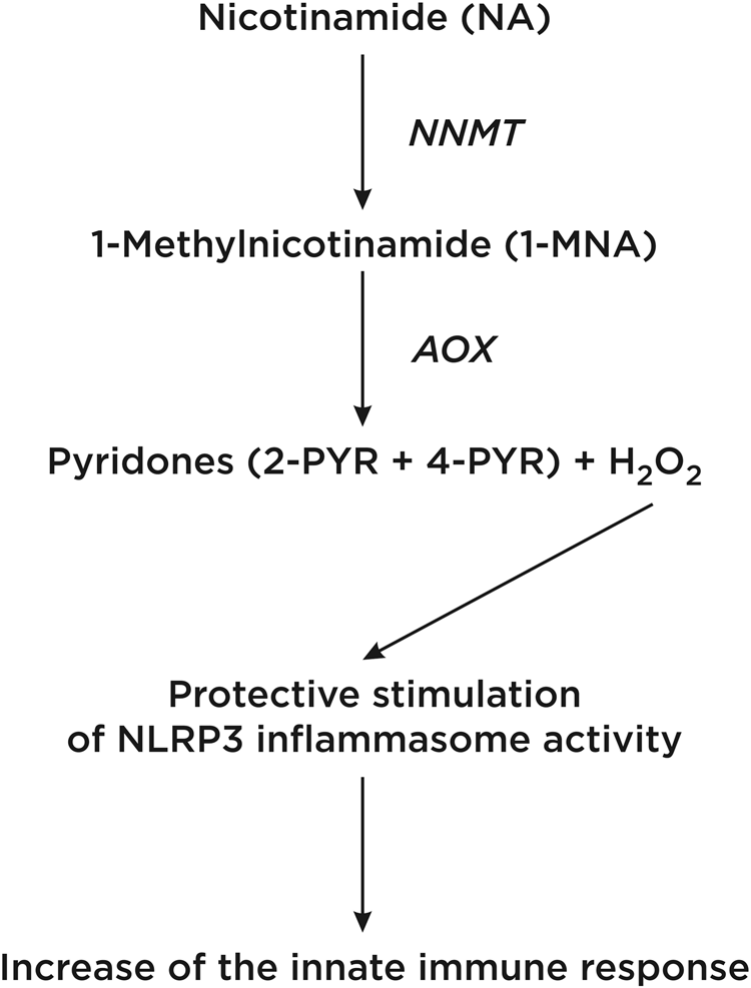
Mitohormetic concept of immune response stimulation by 1-MNA. *NNMT*—nicotinamide N-methyltransferese and *AOX*—aldehyde oxidase.

It is well-known that nicotinamide (NA) is metabolized to 1-methylnicotinamide (1-MNA) via nicotinamide N-methyltransferase (*NNMT*). 1-MNA can be further metabolized to pyridones (2-PYR and 4-PYR) via aldehyde oxidase (*AOX*). All NA metabolites are excreted with urine. The enzymatic formation of pyridones from 1-MNA is associated with the generation of hydrogen peroxide (H_2_O_2_). The process of NA metabolic conversion is presented in the upper part of the scheme shown in Fig. 1.

The formation of H_2_O_2_ is usually associated with oxidative stress, and its presence in tissue is often considered to be negative. On the other hand, pro-oxidative species such as H_2_O_2_ can be beneficial in small amounts, and this effect is known as mitohormesis. It has been shown that 1-MNA promotes *C. elegans* longevity via the mitohormetic mechanism linked to H_2_O_2_ formation by *AOX*^2^.

*AOX* has wide cellular distribution, and its activity is particularly high in the liver, lungs, kidneys, and some endocrine tissues^3^. In respiratory tissues, *AOX* activity is the highest in bronchi.

The protective effect of NLRP3 inflammasome activated by H_2_O_2_ has been nicely documented in a mouse model of septic shock^4^. Surprisingly, the survival rate of glutathione peroxidase 1 knockout (GPx1−/−) mice was much higher than that of wild-type mice. These findings demonstrate that, contrary to much current thinking, early intervention targeting NLRP3 inflammasome activity can induce timely and efficient activation of the innate immune response during acute infection. Clearly, this observation can be linked directly to the mitohormetic concept.

1-MNA, previously regarded as a useless metabolite of NA excreted with urine, has been shown to possess significant anti-inflammatory properties^5^. The pharmacological properties of 1-MNA are quite numerous, and have been documented for many diseases and disorders^6^. The mitohormetic concept of anti-inflammatory activity by 1-MNA is presented in Fig. 1.

As *AOX* expression is particularly high in respiratory tissues, it may be expected that there would be significant 1-MNA anti-inflammatory activity in the airways as well. Indeed, the excretion of 1-MNA with urine has been found to be significantly reduced in respiratory syncytial virus (RSV) infection^7^. It has been suggested that the weakened ability to fend off inflammation during RSV infection is likely due to lower levels of 1-MNA^7^.

Taking all of the above into consideration, the use of Vitamin B3 to prevent inflammation damage associated with COVID-19 seems rational^1^. However, a better effect is likely to be achieved by direct application of 1-MNA. The lower levels of 1-MNA observed in some airway diseases, including viral infections, may further suggest that 1-MNA plays an important physiological role in regulation of the innate immune response.
